# Factors associated with anemia in young children in Brazil

**DOI:** 10.1371/journal.pone.0204504

**Published:** 2018-09-25

**Authors:** Lara Livia Santos da Silva, Wafaie Wahib Fawzi, Marly Augusto Cardoso, ENFAC Working Group

**Affiliations:** 1 Department of Nutrition, School of Public Health, University of São Paulo, São Paulo, São Paulo, Brazil; 2 Department of Global Health and Population, Harvard T.H. Chan School of Public Health, Boston, Massachusetts, United States of America; Pennsylvania State University College of Medicine, UNITED STATES

## Abstract

**Background:**

Anemia is recognized as a major public health problem in childhood, especially in children under 24 months of age. Despite improvements in public health strategies to prevent and control anemia in Brazilian young children in the last decade, few studies have assessed the predictors for this condition in primary health care. Thus, this study aimed to assess the associated factors of anemia in young children who visited primary public health care facilities in Brazil.

**Methods:**

A cross-sectional study was conducted with 520 children aged 11 to 15 months who visited the primary health care in four Brazilian cities. Anemia was defined as hemoglobin concentration < 110 g/L in venous blood samples. Multilevel Poisson regression models were used to describe the associations between anemia and independent variables.

**Results:**

The frequency of anemia was 23.1%. A higher frequency was observed in children who live with more than one other child younger than 5 years in the house (Prevalence Ratio [PR] 1.47; 95% Confidence Interval [CI] 1.01–2.14), who started to receive fruits and vegetables after 8 months of age (PR 1.92; 95% CI 1.19–3.10), who were stunted (PR 2.44; 95% CI 1.32–4.50), who were hospitalized at least once in their life (PR 1.55; 95% CI 1.03–2.33) and who were in the lower tertile of serum folate concentration (PR 2.24; 95% CI 1.30–3.85).

**Conclusions:**

Inadequate complementary feeding practices and morbidity were the main predictors for anemia in early childhood in this population. Improvements in current strategies to promote healthy complementary feeding along with better control of morbidities are recommended to reduce anemia in Brazilian young children.

## Introduction

Anemia refers to a condition in which the blood hemoglobin concentration is lower than normal [1], resulting in poor cognitive and motor development in children and loss of work productivity in adulthood [2, 3].

Iron deficiency is the most common cause of anemia. It is estimated that about 50% of anemia cases are attributed to this micronutrient deficiency [3], although this proportion probably varies substantially across regions and countries [4]. Other causes of anemia include other micronutrient deficiencies, such as folic acid, vitamin A and vitamin B12 deficiencies; the presence of infectious diseases and genetic hemoglobin disorders [2].

Worldwide, it is estimated that 1.62 billion people have anemia, which represents one-quarter of the world’s population, with a higher prevalence in developing countries (9.1%, 25.7% and 42.8% in high, medium and low income countries, respectively). Preschool children, pregnant and non-pregnant women are the three groups most at risk of anemia [5].

In 2011, it was estimated that 273.2 million children aged 6 to 59 months had anemia, which represents 42.6% of this population globally. Africa is the region with highest prevalence of anemia in preschool children (62.3%), followed by the Southeast Asia region (53.8%) and the Eastern Mediterranean region (48.6%) [6].

In Latin America and the Caribbean, a systematic review conducted in 2014 showed that the lowest prevalence rates of anemia among children under 6 years of age were found in Chile and Costa Rica (4.0%), Argentina (16.5%) and Mexico (19.9%), while in Nicaragua, Brazil, Ecuador, El Salvador and Honduras anemia was a moderate public health problem, with prevalence ranging from 20.1% to 37.3%. Guatemala, Haiti, and Bolivia had the highest prevalence rates among children, ranging from 47.7% to 61.3%, indicating a severe public health problem [7].

Specifically in Brazil, the latest National Health Survey to assess the prevalence of anemia in children was conducted in 2006–2007 and revealed that 20.9% of children under 5 years old had anemia [8]. After this period, only small studies were conducted to evaluate this outcome in Brazilian children. Additional studies across the country are needed to identify the prevalence of anemia in this age group.

Studies have shown that children under 24 months of age are at high risk for anemia [9,10]. This can be explained by the rapid growth in this age group with a consequently increased demand for iron and other micronutrients as well as a higher susceptibility to infections [9,11]. Several longitudinal studies have been linked iron deficiency and iron-deficiency anemia to poor cognitive, socio-emotional and psychomotor development among these children, which can produces long-lasting and irreversible effects on their development, even if the iron deficiency has been corrected [12].

There is a lack of data in the literature that identifies the risk factors for anemia in children younger than 2 years of age, but socioeconomic and environmental factors seem to be closely associated with anemia in these children [9, 13, 14].

Given the importance of the crucial period from conception to the first 2 years of life—the first 1000 days—to prevent adverse effects later in the life course [15], it is important to systematically investigate the predictors for anemia in early childhood. In addition, focusing on children assisted at primary health care facilities in different regions of the country is relevant because it is an important place to develop actions for prevention and control of anemia in childhood. Thus, this study aimed to assess the predictors of anemia in young children attending primary health care in four Brazilian cities.

## Materials and methods

### Study design, settings and participants

This cross-sectional study is part of the “*Estudo Nacional de Fortificação caseira da Alimentação Complementar*” (ENFAC Study), which was previously reported [16]. Briefly, the ENFAC Study is a pragmatic, controlled clinical trial conducted in four cities (Rio Branco, Olinda, Goiânia and Porto Alegre) from different regions of Brazil. The ENFAC study was designed to evaluate the impact of multiple micronutrient powder on anemia and nutritional deficiencies of children who visited primary health care.

For the main study, a sample size of at least 105 children in study group (control and intervention groups) for each city was necessary. Considering an additional 30% to cover for possible withdrawals and refusals, 540 children were expected for each group considering the four cities studied. The eligibility criteria of the ENFAC study were parental approval to participate in the study and not currently receiving treatment for anemia. The exclusion criteria were children who were born prematurely (< 37 weeks’ gestation), twins, had reported cases of HIV infection, malaria, tuberculosis or genetic hemoglobin disorders, and fever (> 39° C) on the day of blood sampling. Overall, a total of 1225 children were recruited for the ENFAC study, of whom 1213 were eligible (12 were excluded because of prematurity). For the present analysis, the study population was composed of all children from the control group, aged 11–15 months, and receiving routine pediatric care at 24 large primary health care centers. For this group, 543 children were invited to participate; parents of 22 children (4.1%) declined participation and one child did not provide a blood sample, resulting in a final sample of 520 children. Assuming that the prevalence of anemia in children aged 6–24 months is 40.0% [10], this sample size allowed the detection of the prevalence of anemia with 90% power and a significance level of 0.05.

Written informed consent was obtained from the caregivers of all children after they had been informed of the objectives, benefits and possible risks of the study. All procedures were performed in accordance with ethical standards, and the study was approved by The Human Ethical Review Board of the School of Public Health, University of São Paulo, Brazil.

### Data collection

From June 2012 to January 2013, caregivers of children visiting primary health care centers were invited to participate in this study. During enrollment, trained fieldworkers applied a structured questionnaire through a face-to-face interview with each child’s caregiver, which contained information on socioeconomic, environmental and demographic characteristics of the child, as well as information on child’s birth, maternal data, infant feeding practices, supplementation and morbidities.

After the interview, the length of each child was measured by trained research assistants using standardized procedures and calibrated equipment. Children were measured in dorsal decubitus, on a flat surface, using portable infant measuring boards (model ES-2000, Sanny, Los Angeles, USA). Each measurement was repeated, and the mean value was calculated. Acceptable measurements where those in which the maximum length variation was 0.5 cm. Z-scores of length/height-for-age (HAZ) were calculated according to the World Health Organization Child Growth Standards [17] and those with Z-scores ≥4 or ≤-4 were excluded from the analysis. Stunting was defined as HAZ of < -2 [18].

Fasting venous blood samples were collected by properly trained technicians up to a week after the interview. It was collected in the morning on a day previously scheduled with the caregivers. At the field laboratory, hemoglobin concentration was determined at the time of blood collection by a portable hemoglobinometer (Hb301; HemoCue, Angelholm, Sweden). A separate blood sample was protected from light and centrifuged within 1 hour of collection. After centrifugation, serum and plasma samples were separated in microtubes and frozen at -20ºC before being shipped to the Laboratory of Human Nutrition in the School of Public Health, São Paulo, on dry ice and maintained at -70ºC until further analysis.

In São Paulo, plasma ferritin and soluble transferrin receptor (sTfR) concentrations were measured using commercially available enzyme immunoassays (Ramco, Houston, TX, USA). Serum concentrations of retinol were measured by High Performance Liquid Chromatography (HPLC) methods (HP-1100 HPLC system, Hewlett Packard, Palo Alto, California, USA) as previously described [15], and serum folate concentrations were measured using commercial fluoroimmunoassays (PerkinElmer, Wallac Oy, Turku, Finland). C-reactive protein (CRP) and alpha-1-acid glycoprotein (AGP) in plasma were determined using an IMMAGE Immunochemistry System (Beckman Coulter, Brea, CA, USA).

Anemia was defined as hemoglobin concentration < 110 g/L as established by the World Health Organization [1]. Mild, moderate and severe anemia was defined as hemoglobin concentration of 100–109 g/L, 70–99 g/L and lower than 70 g/L, respectively [1]. Iron deficiency was defined as plasma ferritin concentrations < 12 μg/L and/or sTfR > 8.3 mg/L [19] and iron deficiency anemia was defined as hemoglobin concentration < 110 g/L with plasma ferritin < 12 μg/L and/or sTfR > 8.3 mg/L. Children with serum retinol concentration < 1.05 μmol/L were considered to have marginal vitamin A status. Serum folate concentrations were analyzed in tertiles. The concentrations of CRP > 5 mg/L and AGP > 1 g/L were considered to indicate the presence of inflammation [20].

Children who were anemic or had any nutritional deficiency diagnosed during the study were referred for treatment to primary health care facilities.

### Statistical analysis

Stata statistical software package 12.0 (Statacorp, College Station, Texas, USA) was used for data analysis. The main dependent variable was anemia and the independent variables were: child’s sex and age; child’s ethnicity (classified according to skin color as used in the Brazilian census); maternal education, age and marital status; water supply, categorized as no public network and public network; drinking water treatment, categorized as inadequate (untreated or chlorinated) and adequate (filtered, boiled or mineral); sanitary sewer, categorized as no public sewage or public sewage; number of children aged less than 5 years living in the child’s house; child’s birth weight; breastfeeding in the first hour of life; duration of breastfeeding; timing of introduction of fruits and/or vegetables, meat and beans, defined as early (< 6 months), adequate (≥ 6 months—< 8 months) and late (≥ 8 months); iron and vitamin A plus D supplementation; stunting; hospitalization at least once in life; presence of diarrhea, fever and wheezing in the past 15 days; previous diagnosis of anemia; presence of inflammation; iron deficiency; marginal vitamin A status and folate in tertiles.

Prevalence ratios (PR) were estimated using multilevel mixed-effects Poisson regression models. For this analysis, a hierarchical conceptual framework model with three levels of determination was used for the selection of independent variables, as proposed by Cardoso et al. [10]. The most distal level included socioeconomic, demographic and environmental characteristics; the intermediate level of birth variables, infant feeding practices and use of supplements; and the proximal level of anthropometric variables, reported morbidity and biochemical indicators. Crude analysis controlling for city (as clustering variable), child’s age and sex were conducted for selecting variables to be tested in multiple models (*P*≤ 0.20). Then, at each level of determination, variables were retained in the model if they were associated with the outcome at *P* < 0.10 or if their inclusion in the model changed the PR by 10% or more. The predictors of anemia were considered if, after the adjustment for potential factors at the same level and hierarchically superior levels, presented *P* < 0.05 in the final model. For all variables with missing data (<5%), missing-value categories were included in the multiple models to keep all participants in statistical data analysis.

## Results

A total of 520 children aged 11 to 15 months were included in this study. The characteristics of the children are shown in Table 1. The mean (standard deviation) age of the participants was 13.5 (1.0) months, 50.6% were male and 76.5% were classified as mixed ethnicity. The mothers of almost 80% of the children had more than 7 years of education. About 5% of the study children were stunted and 14.6% had signs of inflammation.

**Table 1:** Characteristics of Brazilian young children who visited primary health care.

Variables	n (%)^a^ or mean ± SD
Age (months)	13.5 ± 1.0
Sex	
Male	263 (50.6)
Female	257 (49.4)
Ethnicity/Skin color	
White	86 (17.1)
Mixed/Brown	385 (76.5)
Black	32 (6.4)
Maternal education (years)	
< 7	103 (20.3)
≥ 7 - < 11	187 (36.8)
≥ 11	218 (42.9)
Birth weight (g)	
< 2500	29 (5.7)
≥ 2500 - < 3500	332 (64.8)
≥ 3500	151 (29.5)
Breastfeeding duration	
< 6 months	148 (28.7)
≥ 6 months	368 (71.3)
Stunting^b^	25 (4.9)
Presence of inflammation^c^	68 (14.6)

SD, standard deviation.

^a^ Totals differ from the total number of study children because of missing values.

^b^ Defined as HAZ < -2 Z-score.

^c^ Defined as CRP > 5 mg/L and AGP > 1 g/L.

The overall frequency of anemia, iron deficiency and iron deficiency anemia were 23.1%, 37.4% and 10.3%, respectively. Among all children, 15.6% presented mild anemia and only 8 children (1.5%) presented severe anemia (Table 2). Median (interquartile range) hemoglobin concentration of the children was 118 (110–126) g/L.

**Table 2:** Frequency of anemia, iron deficiency, iron deficiency anemia and anemia severity among Brazilian young children who visited primary health care.

Biochemical indicators	Total^a^	n (%)
Anemia	520	120 (23.1)
Iron deficiency^b^	503	188 (37.4)
Iron deficiency anemia^c^	503	52 (10.3)
**Frequency of anemia severity**	520	
No anemia		400 (76.9)
Mild (Hb = 100–109 g/L)		81 (15.6)
Moderate (Hb = 70–99 g/L)		31 (6.0)
Severe (Hb < 70 g/L)		8 (1.5)

Hb, blood hemoglobin concentration.

^a^ Totals differ from the total number of study children because of missing values.

^b^ Defined as plasma ferritin < 12 μg/L and/or sTfR > 8.3 mg/L.

^c^ Defined as hemoglobin concentration < 110 g/L with plasma ferritin < 12 μg/L and/or sTfR > 8.3 mg/L.

Table 3 shows the results of the multiple analyses between the independent variables and anemia. In the crude analysis, more than one child younger than 5 years in the house, introduction of fruits and vegetables after 8 months of age, stunting, signs of current inflammation, marginal vitamin A status and low folate concentration were associated with high risk of anemia in these children. In the multiple analysis, signs of current inflammation and marginal vitamin A status lost association with anemia while more than one child younger than 5 years in the house (PR 1.47; 95% CI 1.01–2.14; p = 0.044), introduction of fruits and/or vegetables after 8 months of age (PR 1.92; 95% CI 1.19–3.10; p = 0.007), stunting (PR 2.44; 95% CI 1.32–4.50; p = 0.005), and low folate concentration (PR 2.24; 95% CI 1.30–3.85; p = 0.003) remained significantly associated with this outcome in this population. Previous hospitalization became statistically associated with anemia only after multiple analysis (PR 1.55; 95% CI 1.03–2.33; p = 0.036).

**Table 3:** Associated factors for anemia in Brazilian young children who visited primary health care.

Variables	n	Crude		Multiple adjustment	
		PR (95% CI)	P-value^a^	PR (95% CI)	P-value^b^
**Distal level of determination**					
Maternal education (years)					
< 7	103	1.62 (1.00–2.62)	0.051	1.45 (0.88–2.39)	0.148
≥ 7 - < 11	187	1.29 (0.83–2.00)	0.256	1.19 (0.76–1.86)	0.435
≥ 11	218	Ref.	-	Ref.	-
Drinking water treatment					
Inadequate	217	1.37 (0.91–2.07)	0.126	1.23 (0.81–1.87)	0.329
Adequate	303	Ref.	-	Ref.	-
Children younger than 5 years in the house					
1 child	379	Ref.	-	Ref.	-
> 1 child	141	1.55 (1.07–2.24)	0.021	1.47 (1.01–2.14)	0.044
**Intermediate level of determination**					
Birth weight (g)					
< 2500	29	1.27 (0.65–2.46)	0.485	1.45 (0.74–2.85)	0.281
≥ 2500 - < 3500	332	Ref.	-	Ref.	-
≥ 3500	151	0.69 (0.44–1.08)	0.103	0.71 (0.45–1.11)	0.132
Introduction of fruits or/and vegetables^c^					
Early	217	1.14 (0.74–1.75)	0.561	1.03 (0.67–1.61)	0.877
Adequate	211	Ref.	-	Ref.	-
Late	90	2.08 (1.29–3.33)	0.002	1.92 (1.19–3.10)	0.007
**Proximal level of determination**					
Stunting^d^					
No	483	Ref.	-	Ref.	-
Yes	25	2.49 (1.38–4.50)	0.003	2.44 (1.32–4.50)	0.005
Hospitalization at least once					
No	396	Ref.	-	Ref.	-
Yes	124	1.39 (0.93–2.07)	0.103	1.55 (1.03–2.33)	0.036
Presence of inflammation^e^					
No	399	Ref.	-	Ref.	-
Yes	68	1.97 (1.27–3.05)	0.003	1.57 (0.97–2.54)	0.065
Iron deficiency^f^					
No	315	Ref.	-	Ref.	-
Yes	188	1.38 (0.91–2.08)	0.129	1.35 (0.88–2.07)	0.163
Marginal vitamin A status^g^					
No	322	Ref.	-	Ref.	-
Yes	172	1.53 (1.05–2.25)	0.028	1.31 (0.86–1.99)	0.208
Folate (nmol/L)					
1 tercil (6.3–32.2)	152	2.54 (1.49–4.33)	0.001	2.24 (1.30–3.85)	0.003
2 tercil (32.4–50.1)	152	1.69 (0.96–2.99)	0.070	1.58 (0.89–2.82)	0.119
3 tercil (> 50.1)	156	Ref.	-	Ref.	-

PR, prevalence ratio; CI, confidence interval.

^a^ *P* values were estimated by multilevel mixed-effects Poisson regression models adjusted for child’s sex and age.

^b^ *P* values were estimated by multilevel mixed-effects Poisson regression models adjusted for child’s sex and age, and the variables from each level of determination associated with the outcome at *P* < 0.10 or if their inclusion in the model changed the PR by 10% or more in each level.

^c^ Defined as early (< 6 months); adequate (≥ 6 months—< 8 months); late (≥ 8 months).

^d^ Defined as HAZ < -2 Z-score.

^e^ Defined as CRP > 5 mg/L and AGP > 1 g/L.

^f^ Defined as plasma ferritin < 12 μg/L and/or sTfR > 8.3 mg/L.

^g^ Defined as serum retinol < 1.05 μmol/L.

Missing observations were included in the analysis by creating missing-values categories.

## Discussion

In this study, 23.1% of children aged 11–15 months who visited primary health care facilities in Brazil were anemic. The main associated factors of anemia in this study were children who lived with more than one child younger than 5 years in the house, those who started to receive fruits and/or vegetables after 8 months of age, were hospitalized at least once in their life, were stunted and those in the lowest tertile of folate concentration.

The frequency of anemia in this study was lower than the prevalence found in other studies conducted in Brazil among children younger than 24 months of age [10, 21,22]. A systematic review conducted with studies published from 1996 to 2006 found that, among Brazilian children who visited health care services, the prevalence of anemia ranged from 55.6% to 65.4% in children under 12 months of age and from 55.1% to 89.1% in children aged 12 to 24 months [23]. A population-based study carried out in 2006–2007 in southern Brazil showed that 76% of children between 18 and 23 months had anemia [21] and a study conducted in Acrelândia (Western Brazilian Amazonia) in 2007, identified anemia in 40% of children aged 6 to 24 months [10]. In 2015, a cross-sectional household survey carried out in Alagoas State (Northeast Brazil) found that 47.9% and 37.2% of children aged 6 to 12 months and 13 to 24 months had anemia, respectively [22].

A possible explanation for the lower frequency of anemia noted in this study may be because of the greater investment and attention of the Brazilian government in actions to improve child health in the last three decades [24]. This hypothesis corroborates with a study based on two cross-sectional household surveys carried out in Northeast Brazil which identified a reduction from 45.1% to 27.4% in the prevalence of anemia in children aged 6 to 60 months between the years 2005 and 2015 [22].

Nevertheless, it is important to highlight that, although the frequency found in the present study was lower than the prevalence found in previously published studies [25], it is still high and of concern because of the severe consequences of anemia in young children [19].

In Brazil, one of the main strategies to prevent and control anemia in young children is promoted by the National Program of Iron Supplementation, created in 2005, that distributes iron supplementation for all children aged 6 to 24 months, and pregnant and lactating women. Although several studies have shown the efficacy of iron supplements to prevent iron deficiency anemia in children [26, 27], the major challenge of this strategy is its low effectiveness because of the side effects of iron supplement intake, often attributed to the gastrointestinal intolerance [27, 28]. In the present study, iron supplementation was not significantly associated with anemia. Unfortunately, we have no data on adherence to the use of ferrous sulfate to try to explain this lack of association, but a study conducted with children between 6 and 12 months of age who visited public health services in Viçosa, Brazil, observed that children who did not receive iron supplements had 2.39 times greater chance of having anemia than those who received these supplements [14].

Other important actions to prevent and control anemia in young children in Brazil is the promotion of exclusive breastfeeding during the first 6 months of life and a healthy and timely introduction of complementary feeding. Although we found no association between duration of breastfeeding and anemia in this study, we found that children who started to receive fruit and/or vegetables after 8 months of age were two times more likely to have anemia than children who received these foods between 6 to 8 months of age. According to Davidsson [29], full-term breastfed infants generally have adequate iron status during the first 4–6 months of life, but after this time, when iron stores have been depleted and the breast milk is no longer sufficient to meet the high demand of this mineral in this age, additional dietary iron needs to be supplied for the rapidly expanding blood volume. Although fruits and vegetables are not the best sources of iron in a diet, several of these foods contain ascorbic acid that improves iron absorption [29, 30]. Thus, actions to promote a healthy and timely introduction of complementary feeding needs to be more thoroughly encouraged in primary health care settings in Brazil to reduce anemia in at-risk children.

In this study, children who were hospitalized at least once in life had higher risk for anemia compared with those who were never hospitalized. In addition, those with a current presence of infection had also higher risk for anemia compared with those who were healthy, although this was only marginally significant (p = 0.065). In Brazil, the main causes of hospitalization in children under 4 years old are respiratory diseases followed by parasitic infectious diseases [31]. According to the literature, infectious diseases are a common cause of anemia in childhood, and anemic children are more susceptible to infectious diseases [9]. Furthermore, the duration and severity of the infection is also highly related to the development of anemia [32]. Infectious diseases are important causes of anemia because they reduce dietary intake and the absorption of nutrients, and deplete body stores of iron and other micronutrients. However, iron deficiency may also increase the risk of infection as iron is necessary for normal immune function, creating a vicious cycle between anemia and infectious diseases [33].

Although not assessed in this study, the presence of parasitic infections is also related with anemia. Infections caused by helminthes can cause anemia due to chronic intestinal blood loss and by reducing appetite and nutrition uptake in the intestine [34], and schistosomiasis can cause anemia via four possible mechanisms that includes iron loss in the feces, splenomegaly leading to sequestration and destruction of erythrocytes, autoimmune hemolysis and inflammation [35]. It’s important to highlight that anti helmintics treatment are available in all primary health care facilities in Brazil [25].

Stunting was also associated with anemia in this study. Although only less than 5% of children presented stunting (defined as HAZ < -2), stunted children had a 2.4 times higher risk for anemia compared with those who were not stunted. This finding is in accordance with other studies published in the literature [9, 13, 25]. According to Woldie et al. [13], this association can be explained by noting three aspects: 1) undernourished children are often anemic, 2) low hemoglobin concentration can compromise linear growth, and 3) the coexistence of other micronutrient deficiencies and stunting may increase the development of anemia by a synergistic association. Thus, actions to prevent stunting in children will also contribute to the reduction of anemia in childhood.

It is estimated that 50% of the cases of anemia is due to iron deficiency [3]. In our study, we found that 43% of anemia was due to iron deficiency, that is very close to the WHO estimates. In multiple analyses, the frequency of anemia among iron deficient children was 35% higher than those with no iron deficiency, but it was only marginally significant. In a crude analysis, marginal vitamin A status was significantly associated with anemia but lost association in multiple analyses. As for the folate concentration, we observed a high risk for anemia among children in the lowest tertile of folate concentration when compared with children in the highest tertile of folate concentration. These findings strengthen the evidence that multiple micronutrient deficiencies are important risk factors for anemia [10, 36].

Based on this evidence and aiming to reinforce current strategies to prevent and control anemia in childhood, the Ministry of Health of Brazil launched in 2014 the NUTRISUS (“Multiple Micronutrient in Powder Fortification Strategy”), which consists of the addition of a sachet containing a mixture of vitamins and minerals in powder form to be added to one meal offered daily to children aged 6 to 36 months who visit public day care centers. This strategy is recommended by the World Health Organization in settings where the prevalence of anemia in children aged under 2 years or under 5 years is 20% or higher and is based on a body of evidence that has shown that this strategy was efficient for reducing anemia by 26% and iron deficiency by 52% among young children from 6 to 23 months of age [37]. However, since 2016, Brazil's political and economic crises have drastically reduced access to basic goods, and services to vulnerable populations through many primary health programs, exacerbating child morbidity and mortality within the next decade [38].

Even though it is not the focus of this study, there are some evidences showing a relationship of maternal iron deficiency during pregnancy and lactation and iron deficiency in young children [39]. A study conducted by Kumar et al. [39] indicated that severe maternal iron-deficiency anemia adversely affects cord blood and breast milk iron status, which could have serious consequences for the young infant at a time when iron demands are high. In addition, evidence suggests that maternal iron deficiency compromises maternal cognition and interactive behaviors, which may influence the care of the child [12]. For this reason, it is essential to ensure adequate maternal nutritional status during this important window of opportunity that favours adequate development of young children.

There are some limitations to our study. First, the cross-sectional nature of this study is a potential limitation because this kind of study does not allow one to infer causality and results must thus be interpreted carefully. Furthermore, the use of a sample of users of the health care centers does not allow the extrapolation of results to the general infant population of Brazil. Despite that, it is important to mention that this study is one of the most comprehensive studies on anemia in young children carried out in different regions of the country over the past decade. Additionally, this study was conducted with children attending primary health care, important place for planning actions for prevention and control of anemia in childhood.

## Conclusions

Anemia was found to be a moderate public health problem in Brazilian young children who visited primary health care facilities. More than one child younger than 5 years in the house, introduction of fruits and/or vegetables after 8 months of age, stunting, previous hospitalization and the lowest tertile of folate concentration were associated with high risk for anemia. Improvements to the current strategies for preventing and controlling anemia in Brazil, along with better control of morbidity, are recommended for reducing anemia in children who visit primary health care in this country.
